# REVIO: Range- and Event-Based Visual-Inertial Odometry for Bio-Inspired Sensors

**DOI:** 10.3390/biomimetics7040169

**Published:** 2022-10-18

**Authors:** Yingxun Wang, Bo Shao, Chongchong Zhang, Jiang Zhao, Zhihao Cai

**Affiliations:** 1 Institute of Unmanned System, Beihang University, Beijing 100191, China; 2 School of Automation Science and Electrical Engineering, Beihang University, Beijing 100191, China

**Keywords:** visual-inertial odometry (VIO), range sensor, event camera, sensor fusion

## Abstract

Visual-inertial odometry is critical for Unmanned Aerial Vehicles (UAVs) and robotics. However, there are problems of motion drift and motion blur in sharp brightness changes and fast-motion scenes. It may cause the degradation of image quality, which leads to poor location. Event cameras are bio-inspired vision sensors that offer significant advantages in high-dynamic scenes. Leveraging this property, this paper presents a new range and event-based visual-inertial odometry (REVIO). Firstly, we propose an event-based visual-inertial odometry (EVIO) using sliding window nonlinear optimization. Secondly, REVIO is developed on the basis of EVIO, which fuses events and distances to obtain clear event images and improves the accuracy of position estimation by constructing additional range constraints. Finally, the EVIO and REVIO are tested in three experiments—dataset, handheld and flight—to evaluate the localization performance. The error of REVIO can be reduced by nearly 29% compared with EVIO in the handheld experiment and almost 28% compared with VINS-Mono in the flight experiment, which demonstrates the higher accuracy of REVIO in some fast-motion and high-dynamic scenes.

## 1. Introduction

Location or state estimation is a fundamental and critical problem in areas including Unmanned Aerial Vehicles (UAVs), robotics and autonomous driving [1,2,3]. Global Navigation Satellite System (GNSS) in outdoor non-obstructed environments can provide global, drift-free positioning data. Various sensors can be deployed in autonomous vehicles for high-precision sensing and location. While in GNSS-denied environments such as indoors, buildings and jungles, Visual-Inertial Odometry (VIO), composed of cameras and an Inertial Measurement Unit (IMU), can play an important role for small UAVs, Augmented Reality/Virtual Reality and other light and small equipment which neither have external localization sources nor can carry sensors of larger size and weight such as Light Detection and Ranging [4,5].

VIO can be divided into loosely coupled and tightly coupled according to the fusion pattern. Additionally, the tightly coupled approach is more widely used than the loosely coupled approach. Although the tightly coupled approach increases the dimensionality of variables and computational effort, the association and constraints between data improve the accuracy and enhance the robustness in different scenes. Tightly coupled approaches can be further divided into filter-based and optimization-based methods. The optimization-based approach mainly relies on image processing for feature extraction and optimization of image alignment, such as Open Keyframe-based Visual-Inertial SLAM (OKVIS) [6], Visual Inertial Navigation System (VINS) [7] and ORB-SLAM2/3 [8,9]. The filter-based approach updates the IMU prediction by visual observation to achieve efficient estimation. Multi-State Constraint Kalman Filter (MSCKF) [10] is the most classical filter-based algorithm, in addition to some algorithms under the extended Kalman filter framework such as Robust Visual Inertial Odometry (ROVIO) [11] and Open-VINS [12]. However, there are many high-dynamic scenes such as sharp lighting changes and fast motion in UAVs, robotics and other applications. The image quality can be degraded by motion blur and exposure, resulting in lower estimation accuracy. In addition, under constant acceleration, the IMU cannot be effectively excited. The VIO cannot obtain accurate scale observation information, leading to serious motion drift [13].

Event cameras are bio-inspired sensors developed in the last decade that asynchronously output a high-frequency address-event stream. An event is generated when the luminance change of each pixel exceeds a set threshold. Compared with conventional frame cameras, event cameras have low latency (microsecond) and high dynamic range (140 dB) [14]. Conventional cameras obtain visual information at a constant speed called frames. It outputs an image recording all the motion information at regular intervals. In fast-motion scenes, the pixel value information is limited by the frame rate constraint, and the exposure time cannot match the motion. It results in motion blur in the image and affects localization. Motion causes changes in luminance, and the event camera senses changes in the brightness of pixels on a microsecond scale. It means that the event camera can obtain all motion information as soon as the movement occurs. Especially for high-speed motion, it does not cause motion blur due to the frame rate limitation, which has the potential advantage for application in highly dynamic scenes. However, the different working mode and data type compared to frame cameras make the traditional visual SLAM algorithm cannot be directly used in the event camera.

Due to the limited information carried by each event and the susceptibility to noise, it is difficult to estimate the system state directly. Therefore, early studies mainly used the Bayesian filter-based approaches to update the system state asynchronously through the event generation mechanism [15,16,17]. In addition, there are also ways to package the event streams into groups for processing. An event-based visual odometry (EVO) is proposed in [18] as an event-based visual odometry method capable of running in real-time on the central processing unit. The algorithm constructs a semi-dense map using the estimated poses and events through the spatial scanning method while updating the poses with the edge map formed by the accumulation of events and map matching. To improve the robustness of localization, IMU can be fused to form the event-based visual-inertial odometry. In [19], the events in spatio-temporal windows are cumulatively synthesized into event images after motion compensation, and then feature extraction and tracking are performed. Finally, the tracking of feature points and IMU data are fused to solve the camera trajectory and sparse feature point maps by the keyframe-based nonlinear optimization method. Based on [19,20] proposed an approach fusing image frames, events and IMU to combine the respective advantages of events and images. However, there are a few studies on event-based visual inertial odometry. Additionally, the event-based feature tracking and data association algorithm still suffer from the short tracking time compared with traditional methods. Research is necessary to take full advantage of event cameras to suppress drift and accurately estimate the position in high-dynamic motion scenes.

Range sensors can measure distances with centimeter-level errors over tens of meters, whose light weight and small size can complement vision-inertial modules without significantly increasing the load on the system. Therefore, the algorithm of fusing VIO with range sensors is investigated in some papers. The NASA mars helicopter is equipped with a range-visual-inertial localization system [21], which implements a lightweight algorithm to fuse range information to ensure scalability. However, the algorithm assumes that the ground is flat and consistent, which limits the application scenarios of the algorithm. The work in [5] assumes that the measurement area is a plane perpendicular to the measurement direction and uses ultrasonic ranging to recover the visual scale information based on the assumption. In [22], the scene is further relaxed to arbitrary structures where constraints are constructed for the depth of visual feature points in the VIO using one-dimensional range sensor measurements in the framework of the extended Kalman filter. The central assumption is that the range measurement point and the nearest three visual feature points are considered to be in the same plane. However, this assumption also has limitations and does not apply to stepped scenes with discontinuous depths. Although the scene assumptions in the above papers have limitations, the localization effectiveness of the algorithm is significantly improved with the incorporation of range information.

In this article, we present a new range and event-based visual-inertial odometry (REVIO) for bio-inspired sensors to achieve more stable and accurate localization in high-dynamic scenes with high speed and sharp brightness changes. The main contributions of this paper are as follows:

1. An event-based visual-inertial odometry (EVIO) algorithm is proposed to achieve the location in high-speed motion. Additionally, it is tested on the publicly available event camera dataset.
2. A new visual-inertial odometry REVIO simultaneously fusing range and event. It can improve the accuracy and robustness of the position estimation in typical high-dynamic scenes such as weak textures, fast motion or drastic light changes. The algorithm is validated in handheld experiments.
3. The REVIO algorithm is tested in an actual environment and applied to the flight localization of an UAV.

The remainder of the paper is organized as follows. In Section 2, the preliminaries are introduced. In Section 3, the framework of REVIO fusing range and event is introduced in detail, including a new event-based visual-inertial odometry using sliding window nonlinear optimization, and the fusion of range. In Section 4, three different experiment results and discussions are presented. Section 5 summarizes the contribution of this paper and presents future work.

## 2. Preliminaries

In this section, we introduce the notation that we will use throughout the rest of the paper. We also introduce the event data and IMU model.

**Coordinate Frame**. A point P represented in a coordinate frame A is written as $p^{A}$. A transformation between coordinate frames is represented by a homogeneous matrix $T_{A}^{B}$ that transforms points from frame A to frame B. Its rotational and translational parts are expressed as rotation matrix $R_{A}^{B}$ and translation matrix $t_{A}^{B}$, respectively. This paper mainly involves four coordinate frames: world frame, IMU frame, camera sensor frame and range sensor frame. The sensor body is represented relative to an inertial world frame W. Inside it, we distinguish the camera frame C and the IMU-sensor frame B. An extrinsic calibration of the camera + IMU system must be performed to obtain $T_{C}^{B}$. The range sensor frame is R.

**Event Data**. Event cameras are bio-inspired sensors that work similarly to the ganglion cells in mammal retinae. It asynchronously outputs the information called “event” containing three types of information: the pixel coordinates of the event, the trigger time, and the polarity (the signal of the luminance change) information, expressed as:

(1) e=[u t p]

where $u=\left[\begin{array}{cc} u_{x} & u_{y} \end{array}\right]$ is the event location on the image plane and p is the polarity.

**IMU Model**. IMU kinematic model [23] is as follows:

(2) $$\begin{array}{c} p_{B_{i+1}}^{W}=p_{B_{i}}^{W}+v_{B_{i}}^{W}\Delta t+\underset{t\in [t_{i},t_{i+1}]}{\iint }[R_{B_{t}}^{W}(a_{t}-b_{a_{t}})-g^{W}]dt^{2}\\ v_{B_{i+1}}^{W}=v_{B_{i}}^{W}+\underset{t\in [t_{i},t_{i+1}]}{\int }[R_{B_{t}}^{W}(a_{t}-b_{a_{t}})-g^{W}]dt\\ q_{B_{i+1}}^{W}=\underset{t\in [t_{i},t_{i+1}]}{\int }q_{B_{i}}^{W}⊗\left[\begin{array}{c} 0\\ \frac{1}{2}(\omega_{t}-b_{g_{t}}) \end{array}\right]dt \end{array}$$

where $g^{W}$ is the gravity vector in world frame. $p_{B_{i}}^{W}$, $v_{B_{i}}^{W}$ and $R_{B_{t}}^{W}$ are the position, velocity, and rotation of the IMU frame relative to the world frame in the $i^{th}$ frame. $q_{B_{t}}^{W}$ is the quaternion of $R_{B_{t}}^{W}$ and ⊗ represents quaternion multiplication. $a_{t}$ and $\omega_{t}$ are the measured values of acceleration and angular velocity. $b_{a_{i},g_{i}}$ are the bias of sensors.

## 3. Range and Event-Based Visual-Inertial Odometry (REVIO)

### 3.1. Framework

**Figure 1:**
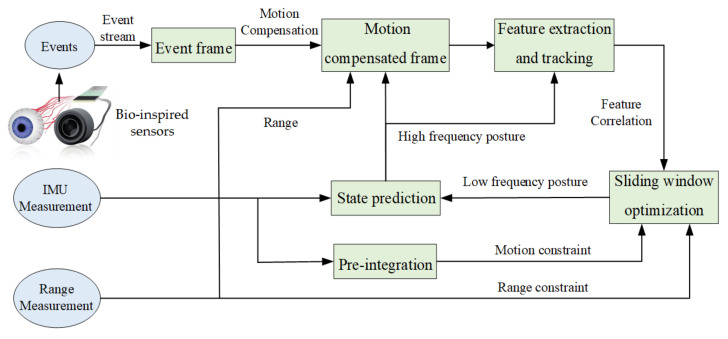
Overview of the proposed pipeline.

The REVIO pipeline is classically composed of two parallel threads. The front-end fuses event, IMU and range information to obtain event images for visual feature point detection and tracking. The back-end constructs an optimization problem using the constraints from the front-end to obtain the state estimation. The framework of our proposed pipeline detailing all steps is illustrated in Figure 1.

The front-end implements pre-processing of various sensor data, including range, event stream and IMU. Firstly, state prediction is performed by IMU, and the image depth is estimated from the range information. Secondly, motion compensation is performed on the event stream to synthesize event images with clear textures. Finally, corner point extraction and optical flow tracking are performed on the event image, during which the IMU data between two frames are pre-integrated and the image interpolation for each frame is matched with the range measurement data at the corresponding moment.

The back-end is a nonlinear sliding window optimization. A fixed number of key frames are maintained within the window. A nonlinear optimization problem on pose, velocity, feature point inverse depth, and IMU bias is constructed to estimate the system state using the visual correlation, IMU pre-integration, range constraints and marginalized state prior constraints.

We improve the method proposed in [19] and integrate range observations into the improved approach for a new VIO fusing range and event. We will present them in the following parts.

### 3.2. EVIO Using Sliding Window Nonlinear Optimization

#### 3.2.1. Front-End of Motion-Compensated Event Frames

The front-end is a pre-processing of the visual observations from the event camera. The data output from the event camera is not image frames and cannot be used directly in traditional image processing. Therefore, the events are first visualized to generate event frames, and then feature extraction and tracking are performed on the images.

(1). Motion Compensation.

The event is triggered by the luminance change. Assuming that the illumination is constant, the luminance change can only come from the relative motion between the camera and the objects in the field of view. The relative motion causes the same pixel to correspond to different areas at different times, so the pixel luminance change also requires grayscale changes of the object. This particular imaging mechanism of event cameras results in them being more sensitive to edge areas. By accumulating a certain number of events, event frame images that reflect edges and textures can be synthesized.

**Figure 2:**
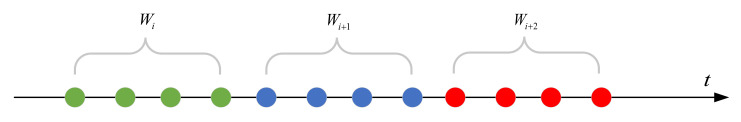
Windows of event stream.

The observed event stream is partitioned into a set of spatio-temporal windows (Figure 2). Each window $W_{i}$ is synthesized into an event frame using the same number of events. The intensity of each pixel on the event image positively correlates with the number of events at that pixel coordinate.

(3) $$f(x)=\underset{i\in W_{k}}{\sum }\delta (x-x_{i})$$

**Figure 3:**
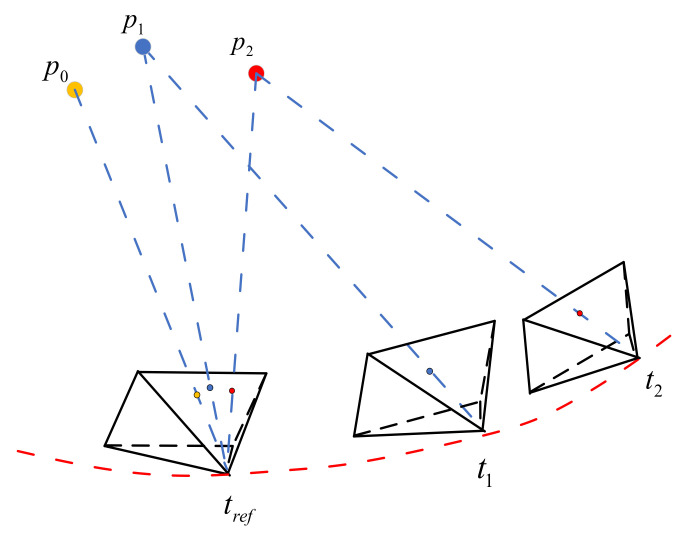
Motion compensation of event stream.

However, each event corresponds to a different timestamp. If the relative motion is fast, direct accumulation of events can produce severe motion blur, which is detrimental to subsequent feature extraction and tracking. Similar to the motion de-distortion of LiDAR point clouds, motion compensation before accumulating event images can reduce motion blur. As shown in Figure 3, events at $t_{1}$ and $t_{2}$ are projected onto the image plane corresponding to $t_{ref}$ by motion compensation.

For the event stream in a period of time, one of the moments is selected as the reference moment $t_{ref}$. Then, the events of all other moments are projected onto the image plane corresponding to the reference moment. For any event $e_{k}$, whose corresponding moment is $t_{k}$, the new coordinate after projection is

(4) $$p_{k}^{\prime }=Ks_{ref}^{-1}T_{ref}^{-1}T_{k}s_{k}K^{-1}p_{k}$$

where *K* is the internal reference matrix of the camera,$T_{k}$ and $T_{ref}$ are the incremental transformation between the camera poses at $t_{k}$ and $t_{ref}$, obtained through integration of the inertial measurements,$s_{k}$ and $s_{ref}$ are the scene depth before and after projection, which is approximated from the average depth of all feature points on the previous event image. The algorithm operates in a planar environment. More accurate depth information can be obtained from other channels, such as range observations and planar constraints, which will be introduced in Section 3.3.

The front-end of the algorithm runs at a higher frequency than the back-end. The frequency of front-end can even exceed 100 Hz. It is decided by the speed of event generation. The timestamp of the newest observation is earlier than that of the latest state at the back end, so the pose cannot be obtained directly from the back end. However, the frequency of IMU is higher than that of the back-end. Based on the latest state of the back-end, a relatively high-frequency, real-time state prediction can be output by integrating the angular velocity and acceleration of the IMU. Then, the position corresponding to each event is obtained by interpolating the timestamp. In this way, we synthesize more clear event frames for image processing.

(2). Feature extraction, prediction and tracking.

Event images are not only related to the environment texture, but also the relative direction of motion between the camera and the environment. In diverse motion patterns, the intensity of textures in different directions can lead to distinct descriptors for the same feature point at different moments. Therefore, we use the strategy of corner point detection plus optical flow method tracking. The actual corner detection is performed with Harris corners. In order to distribute the feature points evenly in each region of the image and improve the accuracy of pose estimation, we divide the image into M × N regions and maintain a finite number of feature points in each region.

For a newly arrived frame, forward optical flow from the previous to the current frame is performed. This paper involves some fast-motion scenes where the feature points move on the image with large amplitude, resulting in poor tracking quality of the optical flow method. To solve the above problem, based on the multilayer optical flow method, we provide predicted values of the coordinate on the next frame for each feature point of the previous frame. For the triangulated feature point *k*, $p_{k_{i+1}}$ is the normalized coordinate of the feature point on the previous frame i. The pose *T_i + 1_* of the current frame *i*+1 is predicted using IMU and projected onto the current frame as follows:

(5) $$p_{k_{i+1}}=s_{k_{i+1}}T_{i+1}^{-1}T_{i}s_{k_{i}}p_{k_{i}}$$

For the untriangulated feature points, different strategies are selected in diverse scenes. The general sceneries are directly set to the coordinates of the previous frame. While for the overhead view scene in this paper, the average optical flow is calculated to get the predicted coordinates of the feature point in the current frame.

After getting the tracking values of the feature points in the current frame, we make another reverse optical flow from the current to the previous frame to ensure the tracking quality. The coordinates of the feature points in the previous frame are calculated in reverse. The tracking is considered successful only when the error between the two calculations is less than the threshold.

In the end, the matching relationship of feature points is used to remove a small number of false matches by solving the fundamental matrix from the previous frame to the current frame based on Random Sample Consensus (RANSAC). Thus, we obtain a more accurate inter-frame correlation of feature points.

#### 3.2.2. Back-End with Sliding Window Non-Linear Optimization

The sliding window optimization with fixed window size is used in the back-end to control the optimized scale and efficiency. The window size is *N*+1, and the optimization variables are

(6) $$\begin{array}{c} \chi =\left[x_{0},x_{1},\ldots ,x_{N},\rho_{0},\rho_{1},\ldots ,\rho_{m}\right]\\ x_{i}=\left[p_{B_{i}}^{W},q_{B_{i}}^{W},v_{B_{i}}^{W},b_{a_{i}},b_{g_{i}}\right],i\in [0,N] \end{array}$$

where $\rho_{k}$ is the inverse depth of the feature point *k* on the starting frame, $p_{B_{i}}^{W}$, $q_{B_{i}}^{W}$ and $v_{B_{i}}^{W}$ are the position, rotation, and velocity of the IMU frame relative to the world frame in the $i^{th}$ frame, $b_{a_{i}}$ and $b_{g_{i}}$ are the biases of the accelerometer and gyroscope. Meanwhile, the extrinsic parameter $\left[q_{C}^{B},t_{C}^{B}\right]$ between IMU and camera and the extrinsic parameter $\left[q_{R}^{C},t_{R}^{C}\right]$ between camera and the range sensor can also be calibrated online as variables.

**Figure 4:**
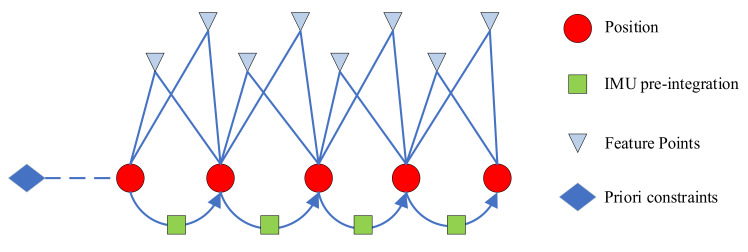
Back-end optimization factors.

To preserve the observation information and constraints carried by the old keyframes, we use the marginalization strategy to transform them into state prior constraints within the window. Thus, the overall cost function of the back-end includes the following three constraints: IMU pre-integration constraints, visual reprojection constraints, and marginalized prior constraints. Figure 4 shows the back-end optimization factors.

(1). IMU pre-integration constraints.

According to the IMU model in Section 2, we can obtain the following equation:

(7) $$\begin{array}{c} p_{B_{i+1}}^{B_{i}}=v_{B_{i}}^{B_{i}}\Delta t-\frac{1}{2}g^{B_{i}}\Delta t^{2}+\alpha_{B_{i+1}}^{B_{i}}\\ v_{B_{i+1}}^{B_{i}}=v_{B_{i}}^{B_{i}}-g^{B_{i}}\Delta t+\beta_{B_{i+1}}^{B_{i}}\\ q_{B_{i+1}}^{B_{i}}=q_{B_{i}}^{B_{i}}\gamma_{B_{i+1}}^{B_{i}} \end{array}$$

where $\alpha_{B_{i+1}}^{B_{i}}$, $\beta_{B_{i+1}}^{B_{i}}$ and $\gamma_{B_{i+1}}^{B_{i}}$ denote the pre-integrated quantities.

(8) $$\begin{array}{c} \alpha_{B_{i+1}}^{B_{i}}=\underset{t\in [t_{i},t_{i+1}]}{\iint }R_{t}^{B_{i}}(a_{t}-b_{a_{t}})dt^{2}\\ \beta_{B_{i+1}}^{B_{i}}=\underset{t\in [t_{i},t_{i+1}]}{\int }R_{t}^{B_{i}}(a_{t}-b_{a_{t}})dt\\ \gamma_{B_{i+1}}^{B_{i}}=\underset{t\in [t_{i},t_{i+1}]}{\int }\frac{1}{2}\Omega (\omega_{t}-b_{g_{t}})q_{t}^{B_{i}}dt \end{array}$$

The pre-integration provides position, velocity and attitude constraints between consecutive frames, and the residuals are constructed as follows:

(9) $$\begin{array}{c} \begin{array}{c} \delta \alpha_{B_{i+1}}^{B_{i}}=R_{W}^{B_{i}}\left(p_{B_{i+1}}^{W}-p_{B_{i}}^{W}+\frac{1}{2}g^{W}\Delta t^{2}-v_{B_{i}}^{W}\Delta t_{k}\right)-\alpha_{B_{i+1}}^{B_{i}}\\ \delta \beta_{B_{i+1}}^{B_{i}}=R_{W}^{B_{i}}\left(v_{B_{i+1}}^{W}-v_{B_{i}}^{W}+g^{W}\Delta t\right)-\beta_{B_{i+1}}^{B_{i}}\\ \delta \theta_{B_{i+1}}^{B_{i}}=2{\left[q_{B_{i+1}}^{W}⊗{q_{B_{i}}^{W}}^{-1}⊗{\gamma_{B_{i+1}}^{B_{i}}}^{-1}\right]}_{xyz}\\ \delta b_{a}=b_{a_{b_{i+1}}}-b_{a_{bi}}\\ \delta b_{g}=b_{g_{b_{i+1}}}-b_{g_{b_{i}}} \end{array} \end{array}$$

(2). Reprojection constraints.

Visual geometric constraints are provided by observing the same feature points in different frames. We use the coordinates of the feature point at the start frame and the inverse depth to represent its 3D coordinates. Each feature point is projected onto the other keyframes using the inverse depth and the pose. The reprojection error is obtained by calculating the difference between the projected coordinates and the observed coordinates of the keyframe. For a feature point *k*, the projection from the $i^{th}$ frame to $j^{th}$ frame is represented as:

(10) $$p_{k_{j}}^{\prime }={\left(T_{C}^{B}\right)}^{-1}{\left(T_{B_{j}}^{W}\right)}^{-1}T_{B_{i}}^{W}T_{C}^{B}\frac{1}{\lambda_{k}}p_{k_{i}}$$

where $p_{k_{i,j}}$ are the observed coordinates of the feature points in the $i^{th}$ and $j^{th}$ frame, ${p^{\prime }}_{k_{j}}$ is the projected coordinate in the $j^{th}$ frame, $\lambda_{k}$ is the inverse depth of the feature point at the starting frame.

The reprojection error is denoted as:

(11) $$e_{k_{ij}}=\left[\begin{array}{c} p_{jx}\\ p_{jy} \end{array}\right]-\frac{1}{p_{jz}^{\prime }}\left[\begin{array}{c} p_{jx}^{\prime }\\ p_{jy}^{\prime } \end{array}\right]$$

(3). Marginalized priori constraints.

To control the dimension of optimization while maintaining the observation or constraint information carried by the old keyframes, the Schur complement is used to transform past states and observations into state prior constraints within the window.

For a nonlinear optimization problem, the nonlinear cost function is linearized in each iterative optimization to transform the nonlinear problem into a linear least squares problem. Taking the Gaussian Newton method as an example, the optimization problem eventually turns into solving the following equation:

(12) Hδx=b

where $H=J^{T}J$, $b=J^{T}r$, J is the Jacobi matrix of residuals *r* about the optimization variables, and δx is the increment of variable x in the iteration.

The variable x is divided into parts that need to be marginalized and others, so δx is

(13) $$\delta x=\left[\begin{array}{c} \delta x_{1}\\ \delta x_{2} \end{array}\right]$$

Correspondingly, the matrices H and b are divided into:

(14) $$H=\left[\begin{array}{cc} H_{11} & H_{12}\\ H_{21} & H_{22} \end{array}\right],\;b=\left[\begin{array}{c} b_{1}\\ b_{2} \end{array}\right]$$

In this case, $\delta x_{1}$ and $\delta x_{2}$ are coupled. The Gaussian elimination method is used to marginalize $\delta x_{2}$ and transform it into an a priori constraint of $\delta x_{1}$.

(15) $$\left(H_{11}-H_{12}H_{22}^{T}H_{21}\right)\delta x_{1}=b_{1}-H_{12}H_{22}^{T}b_{2}$$

### 3.3. Fusing Range and Event for VIO

The improved EVIO still relies on the vision for state estimation. However, in scenes with weak textures or fast motion, the reduced number of visual feature points and shorter tracking lengths can reduce the accuracy of image depth estimation and fail to provide accurate constraint information, leading to increased localization estimation errors. In particular, when the state undergoes constant acceleration motion, VIO exists scale unobservability, and the state estimation drifts. We propose the REVIO algorithm fusing range and EVIO to solve the above problems. The integration of range observation can provide absolute scale information and use the planar structure in the scene to provide constraints for motion estimation and feature point depth estimation to obtain more accurate estimations.

The algorithm in this paper is based on the following assumptions. The direction of the range measurement is defined as the optical axis direction of the camera. The system is mounted on a ground-facing carrier (e.g., a UAV), i.e., the visual information comes from the horizontal plane.

#### 3.3.1. Front-End Correction with Range Sensors

The integration of range observation provides more accurate image depth estimation in the motion compensation of the front-end. All current feature points are assumed to be on the same horizontal plane, and the range information denotes the distance from the sensor to that plane. The coordinates of the range measurement point in IMU frame can be expressed as:

(16) $$p_{j}^{B}=R_{C}^{B}(R_{R}^{C}\left[\begin{array}{c} 0\\ 0\\ r_{j} \end{array}\right]+t_{R}^{C})+t_{C}^{B}$$

where $r_{j}$ is the range observation in the $j^{th}$ frame, $R_{C}^{B}$ and $t_{C}^{B}$ are the rotation and translation external parameters between the camera and IMU, $R_{R}^{C}$ and $t_{R}^{C}$ are the rotation and translation external parameters between the camera and the range sensor.

The distance from IMU to the plane in the $j^{th}$ frame is

(17) $$d_{j}=-n^{T}R_{j}p_{j}^{B}$$

where $R_{j}$ is the rotation of IMU in the $j^{th}$ frame in the world frame and *n* is the unit normal vector of the plane in the world frame.

Therefore, the depth ${\bar{S}}_{k_{j}}$ of the feature point in the plane at the $j^{th}$ frame can be expressed as:

(18) $${\bar{s}}_{k_{j}}=\frac{n^{T}R_{j}R_{C}^{B}(R_{R}^{C}\left[\begin{array}{c} 0\\ 0\\ r_{j} \end{array}\right]+t_{R}^{C})}{n^{T}R_{j}R_{C}^{B}p_{k_{j}}}$$

where $p_{k_{j}}$ is the normalized coordinate of the feature point *k* in the $j^{th}$ frame.

The depth information of feature points obtained by range observation is used for the front-end motion compensation correction to acquire much clearer event images.

#### 3.3.2. Back-End of Adding Range Constraints

Range observation can provide additional constraints for the back-end optimization estimation: ground constraints and generalized scenario constraints. We can obtain more accurate state motion estimation by adding the new constraints.

(1). Ground constraints.

The coordinate of the feature point *k* in the $j^{th}$ frame in world frame can be denoted as:

(19) $$p_{k_{j}}^{W}=R_{i}(R_{C}^{B}\frac{1}{\lambda_{k}}p_{k_{j}}+t_{C}^{B})+t_{i}$$

where $R_{i}$ and $t_{i}$ are the rotation and translation of IMU, $\lambda_{k}$ is the inverse depth of the feature point at the starting $i^{th}$ frame. The feature point is located in the plane, and the distance from IMU to the plane in the $j^{th}$ frame is the inner product of two vectors, which are the line connecting the IMU position to the feature point and the normal of the plane.

(20) $$d_{k_{j}}=-n^{T}(p_{k_{j}}^{W}-t_{i})$$

The range $d_{j}$ from IMU to the plane in the $j^{th}$ frame has been given by (17) through the range observation. $d_{j}$ and $d_{k_{j}}$ should be equal, which means that the line between the feature point and the range observation point is perpendicular to the normal vector of the plane.

(21) $$n^{T}(p_{j}^{W}-p_{k_{j}}^{W})=0$$

The variables included in this constraint are the poses in the frame, and the inverse depth of feature point. External parameters between IMU, camera and range sensors can also be added for online optimization. Each image frame has the corresponding range sensor data. Therefore, each feature point can establish constraints with all observed frames, which is formally consistent with the reprojection error of vision.

(2). Generalized scenario constraints.

**Figure 5:**
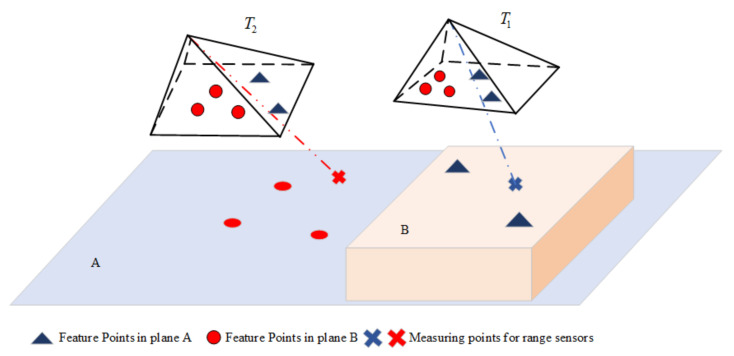
The feature point and range point are not in the same plane at the different moments of T_1_ and T_2_.

The image captured by the camera in the actual scene may not be a complete plane. In Figure 5, the camera observes several points distributed in different planes at different locations. If it is assumed that all feature points and range measurement points belong to the same plane, this will introduce false constraints and lead to a decrease in the accuracy of the back-end state estimation. Therefore, we should determine whether the feature points and the range measurement points are in the same plane.

Determining whether the feature points and range measurement points are in the same plane can be converted to determine whether the depth calculated based on this assumption is reasonable. We can calculate the depth of the feature point in the $j^{th}$ frame by (18). The estimated depth of the feature point *k* in the starting frame is ${\tilde{s}}_{k_{i}}$. The reprojection error of the feature point at two depths is calculated, and the results are compared to determine whether the depth is reliable.

First, we calculate the reprojection error of feature point from the $i^{th}$ frame to the $j^{th}$ frame based on the estimated depth ${\tilde{s}}_{k_{i}}$. The coordinate of the feature point in the $j^{th}$ frame in camera frame is

(22) $${\tilde{p}}_{k_{j}}^{C}={R_{C}^{B}}^{T}(R_{j}^{T}(R_{i}(R_{C}^{B}{\tilde{s}}_{k_{i}}p_{k_{i}}^{c}+t_{C}^{B})+t_{i}-t_{j})-t_{C}^{B})$$

The coordinate is normalized and subtracted to obtain the reprojection error.

(23) $$e_{1}={\tilde{p}}_{k_{j}}^{C}-p_{k_{j}}^{C}$$

Next, the reprojection error is calculated using the depth ${\bar{s}}_{k_{i}}$.

(24) $${\bar{p}}_{k_{i}}^{C}={R_{C}^{B}}^{T}(R_{i}^{T}(R_{j}(R_{C}^{B}{\bar{s}}_{k_{j}}p_{k_{j}}^{c}+t_{C}^{B})+t_{j}-t_{i})-t_{C}^{B})$$

The reprojection error is denoted as:

(25) $$e_{2}={\bar{p}}_{k_{i}}^{C}-p_{k_{i}}^{C}$$

If $\left|e_{2}\right|\leq \left|e_{1}\right|$, it means that the depth estimated by the coplanarity assumption is reasonable, and the feature points belong to the same plane as the range measurement points. $e_{2}$ is more consistent with the current positional constraint than $e_{1}$, and the plane constraint is added to the back-end optimization. Otherwise, the visual reprojection constraints of the feature points are constructed and added to the back-end optimization. In addition, range constraints can be considered to be added in the neighborhood around the range observation point to avoid introducing error constraints and reduce the computational effort.

## 4. Experiments

In this section, we perform three sets of experiments to test the accuracy of our proposed pipeline. Both qualitative and quantitative results are provided, which demonstrate the effectiveness of our method. The first set of experiments is dataset experiments. We evaluate the accuracy of our improved EVIO algorithm on public datasets. The second set of experiments compares REVIO with EVIO to prove the superiority of increased range observation. The third set of experiments further demonstrates the performance of REVIO algorithm through the actual flight.

### 4.1. Dataset Experiments: Our EVIO versus Other Algorithms

We use the Event Camera Dataset [24] to evaluate the accuracy of the proposed pipeline. The Event Camera Dataset contains many sequences captured with a DAVIS-240C camera with ground truth tracking information. Particularly, it contains extremely fast motions and scenes with a very high-dynamic range. The DAVIS sensor embeds a 240 × 180 pixels event camera with a 1 kHz IMU and also delivers standard frames at 24 Hz.

**Figure 6:**
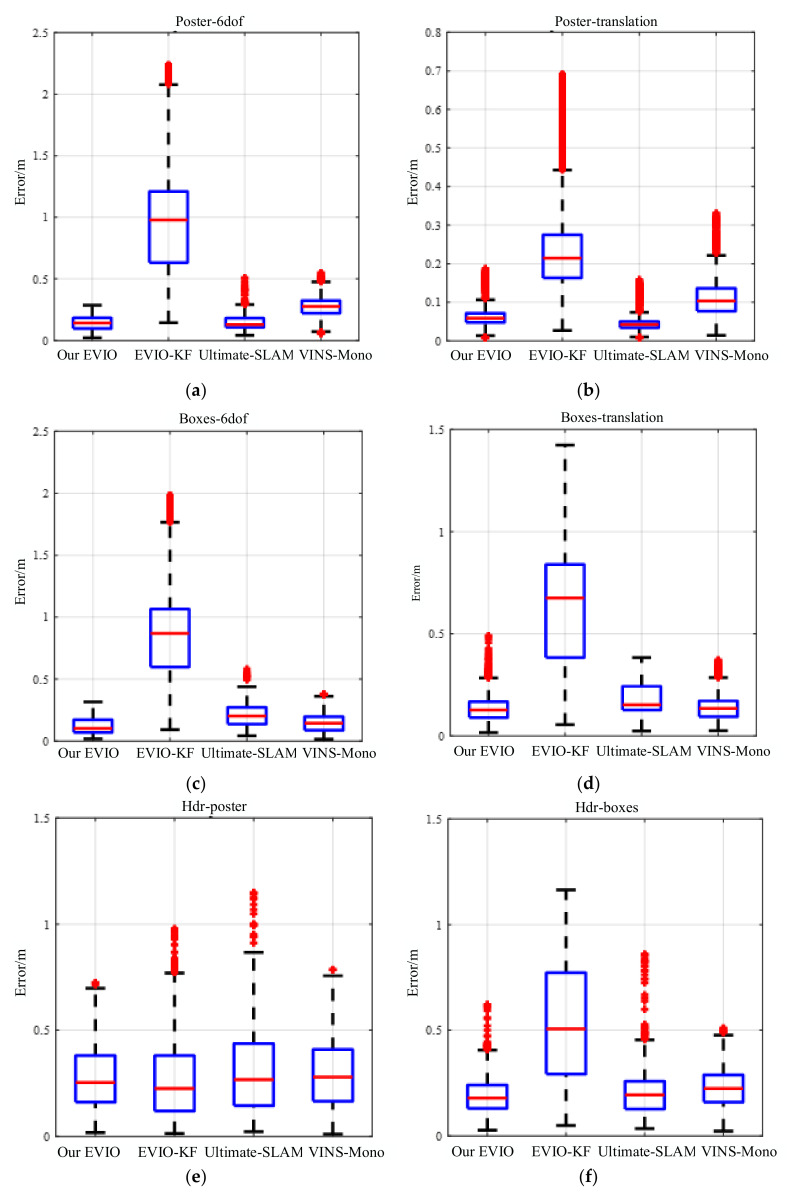
RMSE of proposed pipeline against others in Event Camera Dataset (**a**) poster_6dof; (**b**) poster_translation; (**c**) boxes_6dof; (**d**) boxes_translation; (**e**) hdr_poster; (**f**) hdr_boxes.

To demonstrate the advantages of our EVIO in a highly dynamic environment, we conducted comparative tests on the dataset sequence using different algorithms, including VINS-Mono, EVIO-KF, Ultimate-SLAM, etc. The estimated and ground truth trajectories are aligned with a 6-DOF transformation in SE3 to evaluate the results. Then, we compute the root mean squared error (RMSE) to compare the accuracy of algorithm. Table 1 shows the results obtained when running these algorithms in six different dataset sequences. In addition, in Figure 6, we use the relative error metric proposed in [25], which evaluates the relative error by averaging the drift over trajectories of different lengths.

From the results, we can see that the proposed pipeline outperforms the other three methods on these dataset sequences. Using only events (E) and IMU (I), the accuracy of our method is much better than that of EVIO-KF. The error can be reduced by about 80% on the poster_6dof sequence of six degrees of freedom with strong motion. In contrast to Ultimate-SLAM using images, events, and IMUs, our EVIO achieves comparable or even better accuracy, with an error reduction of about 37% on boxes_6dof sequences. Compared to VINS-Mono using images, the accuracy can improve by nearly 37% on dataset sequences with small scene depth and intense motion.

However, in scenes such as stationary or motion along the optical axis, the signal-to-noise ratio of the event stream can be too low for poor quality of the event image, which affects feature tracking and increases the position estimation error. Traditional images in such scenes provide better constraints, which is the reason why Ultimate-SLAM and VINS-Mono can achieve higher accuracy. In scenes with continuous fast motion and high dynamic range, our EVIO can achieve higher accuracy.

**Figure 7:**
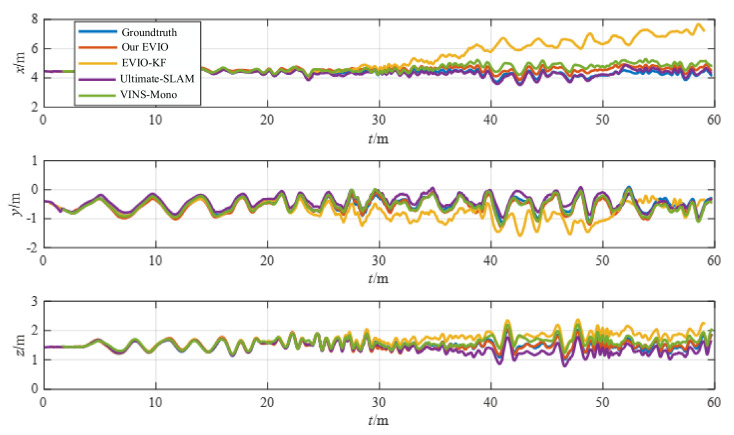
The position estimation of different algorithms for the poster_6dof.

**Figure 8:**
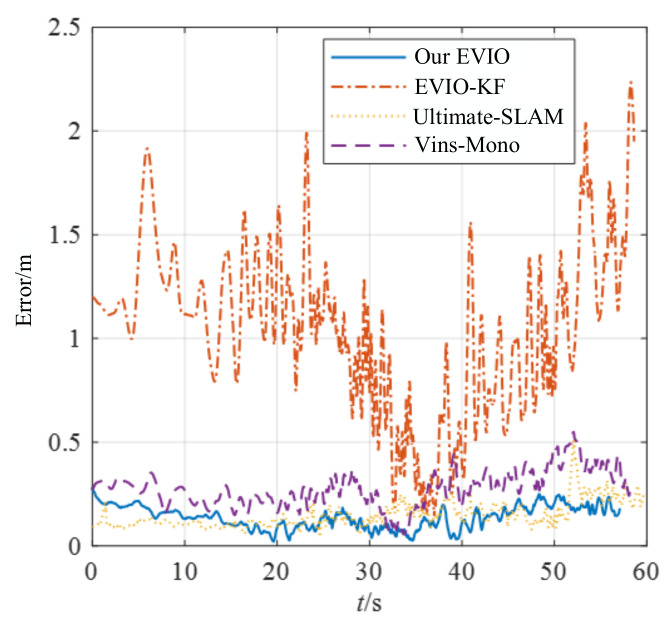
The position error of different algorithms for the poster_6dof.

To further demonstrate the capabilities of our method, we chose one of the dataset sequences for the experiment. For typical scenes with fast translations and rotations, such as the poster_6dof sequence, the trajectories and error distributions estimated by the four algorithms are shown in Figure 7 and Figure 8.

The estimation accuracy of our proposed pipeline is better than that of VINS-Mono and EVIO-KF. Although the accuracy is comparable to that of Ultimate-SLAM, Ultimate-SLAM uses both event streams and images, which is more computationally intensive. In fast-motion scenes, the algorithm in this paper can construct motion constraints more accurately with less computation, and the estimation accuracy is higher.

### 4.2. Handheld Experiments: REVIO versus EVIO

Considering that the current public dataset does not contain range observation data, the dataset experiment cannot reflect the advantage of range, and the scenes of the dataset do not apply to REVIO. To evaluate the properties of REVIO after fusing range, a sensing system consisting of an event camera and a depth camera is constructed to test the accuracy of REVIO in real devices and fast-motion environments through handheld experiments.

**Figure 9:**
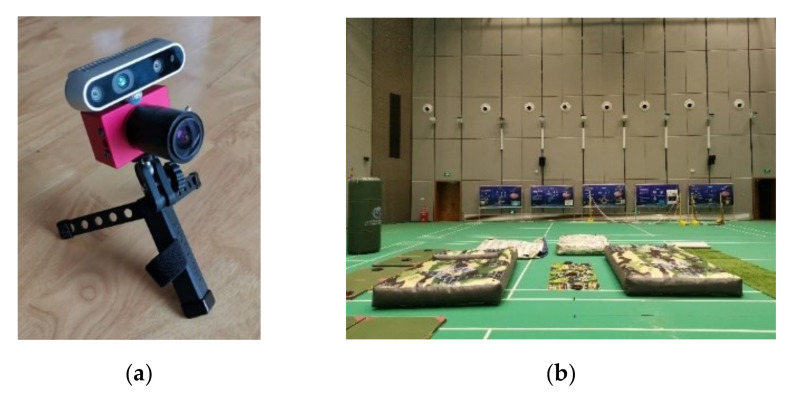
Equipment and scenarios for handheld experiments: (**a**) the handheld device; (**b**) the environment in the hall.

The sensor system for handheld experiments is shown in Figure 9 (a), which consists of an IniVation event camera DAVIS 346 (bottom) and an Intel RealSense Depth Camera D435i (top). The DAVIS 346 sensor embeds a 346 × 246 pixels event camera with a 1 kHz IMU and also delivers standard frames at 24 Hz. The D435i delivers depth images at 30 Hz. We choose the depth of the depth image centroid to simulate the range observation for testing the effect of the addition of range constraints on the performance of the localization algorithm.

**Figure 10:**
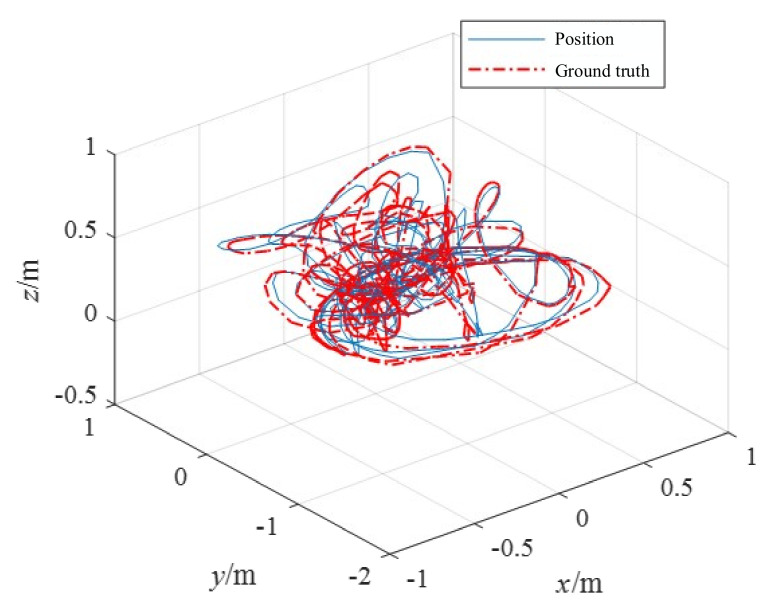
The position estimation of handheld experiments.

**Figure 11:**
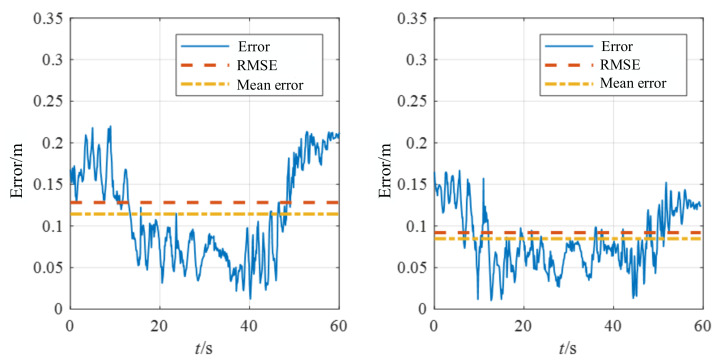
Comparison of the errors using E + I (**left**) with R + E + I (**right**) at maximum speed 3.489 m/s. The left is EVIO using events (E) and IMU (I). The right is REVIO using range (R), events (E) and IMU (I).

The handheld experiments were performed in the experimental hall configured with Optitrack (Figure 9b). The illumination information of the experimental hall is 5 Lux-145 Lux. Optitrack is a motion capture system developed by NaturalPoint Inc for applications including movement sciences, robotics and more. The data obtained from Optitrack is considered the ground truth. The accuracy is evaluated by calculating the relative position error between the estimated trajectory and the Optitrack trajectory. Figure 10 and Figure 11 demonstrate the position estimation of REVIO under the dataset with the maximum speed of 3.489m/s and the remarkable accuracy of REVIO compared to EVIO.

To further demonstrate the capabilities of our method, we present several datasets with different speeds for the experiment. Table 2 provides a comparison of the experiment results performed between sequences of motion datasets at four speeds. At lower speeds, the error of REVIO and EVIO is relatively close to each other. When the speed gradually increases, the error of REVIO is reduced to nearly 29% than that of EVIO. The position estimation accuracy is enhanced after fusing the range constraint, and the performance gap between the two algorithms gradually widens with the increasing speed.

Fast motion produces more obvious motion blur, causing an increase in tracking error. Further, it leads to a decrease in the depth estimation accuracy, the visual part cannot provide effective constraints, and the position estimation produces drift. The addition of range observation provides scale constraints, which depresses the drift and improves the estimation accuracy.

### 4.3. Flight Experiments: REVIO versus VINS-Mono

**Figure 12:**
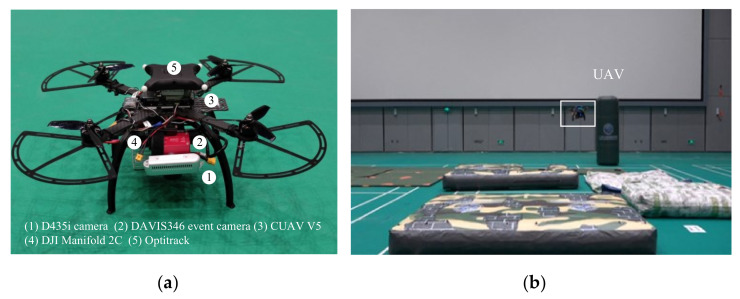
The quadrotor and environment for flight experiments: (**a**) quadrotor platform used for the flight experiments; (**b**) the flying experiment.

In order to show the potential of REVIO in real scenes, we ran our approach onboard an autonomous quadrotor and used it to fly autonomously in fast-motion scenes. As Figure 12a shows, the aerial platform is equipped with a DAVIS 346 event camera and a D435i standard camera. The D435i camera is used to record depth images. Both standard and event cameras are facing downward. The state estimation and the control algorithm are run on a DJI Manifold 2C which contain an i7-8550U CPU running Ubuntu 18.04 and ROS. The motor thrust commands from the control algorithm are sent to motors through a CUAV V5 flight control board. Figure 12b shows the test site equipped with the Optitrack optical motion capture system, whose positioning data is only used as the truth-value for evaluation. In addition, the sensor and Optitrack data during the flight are saved for subsequent offline testing.

**Figure 13:**
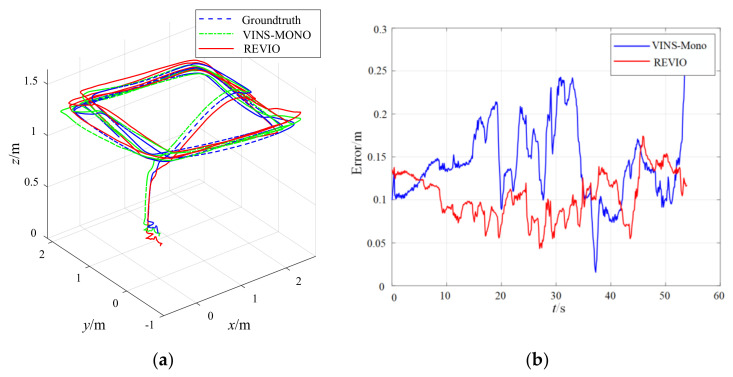
Flying experiment: (**a**) the estimated trajectory of flying experiment; (**b**) the position error in flying experiment.

The UAV achieves autonomous flight in PX4 Offboard mode with the estimated pose from the REVIO algorithm. The comparison between the flight trajectory estimated by REVIO and the truth-value of Optitrack is shown in Figure 13, where the average accuracy can reach about 10 cm.

During the experiment, it was found that the tracking of feature points was not stable in texture-less region, and errors in visual observations occurred, leading to drift in the VINS-Mono positional estimates. Range observations can provide additional scale constraints to compensate for the effects caused by visual tracking instability.

**Figure 14:**
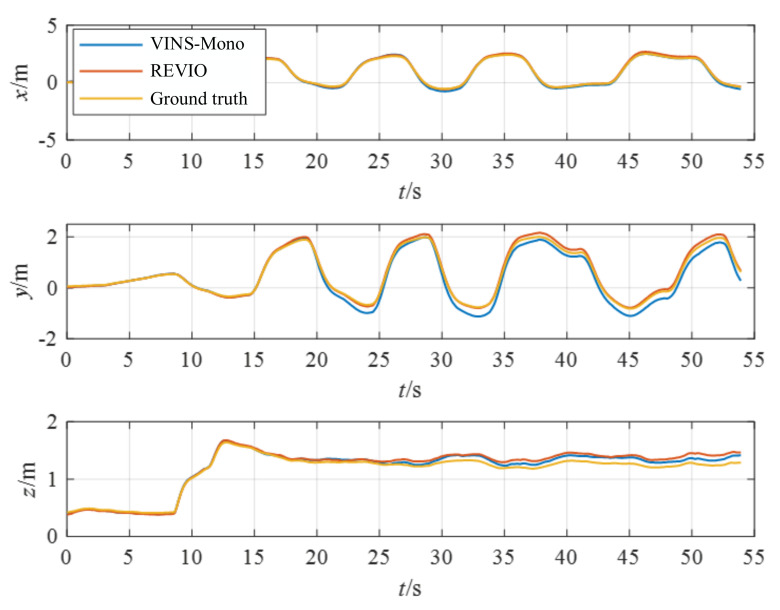
Comparison of REVIO versus VINS-Mono.

**Figure 15:**
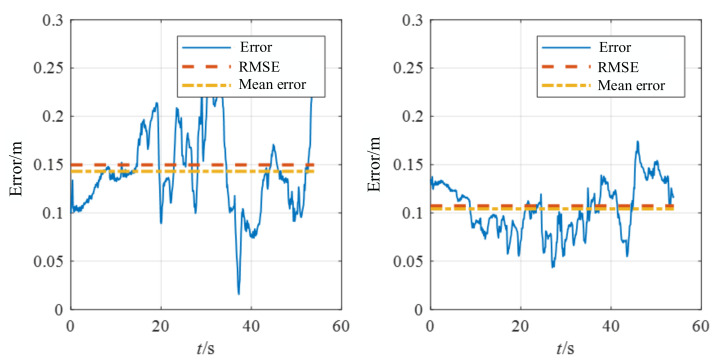
Comparison of the position error between VINS-Mono (**left**) and REVIO (*right*).

Figure 14 and Figure 15 show the position estimation error comparison and estimated trajectory comparison between REVIO and VINS-Mono. The position estimation error of REVIO is smaller than that of VINS-Mono. The average position estimation error of VINS-Mono is 0.148887 m against 0.107473 m for REVIO, which is about 28% less. In addition, the high-frequency vibration of the motor introduces a large amount of noise to the IMU measurements, and the constraints on the range observation scale of the VIO system significantly reduce the position estimation error.

## 5. Conclusions

In this paper, we propose a range and event-based visual-inertial odometry (REVIO) for bio-inspired sensors running in real-time on drones. It constructs a joint cost function to estimate the motion state of the system using event stream, range observations and IMU data. The experiment results show that the integration of range constraints further improves the accuracy and stability of the algorithm in structured environments and highly dynamic scenes and reduces the drift of the system. The average position estimation error of REVIO can be reduced by nearly 28% or more compared with other VIO methods. We also propose an improved EVIO algorithm. The dataset experiment results show that the estimation error of our EVIO algorithm is up to about 80% less compared with other algorithms in high-dynamic scenes with fast motion or drastic illumination changes. However, the method only applies to the coplanar constraint of range observation points and horizontal surface feature points, which is inadequate in terms of constraint. In the future, the integration of multi-plane observation constraints can be considered to provide accurate and robust state estimation in more complex scenes. In addition, the effect of illumination and noise on the algorithm is not considered, which is also worth studying in the next step.

## Figures and Tables

**Table 1:** The root mean squared error (RMSE) comparison between some algorithms.

Sequence	Max Speed (m/s)	Length (m)	RMSE (m)			
			Our EVIO (E + I) *	EVIO-KF [19] (E + I)	Ultimate_SLAM [20] (Fr + E + I)	VINS_Mono [10] (Fr + I)
poster_6dof	3.370	61.143	**0.147**	1.036	0.161	0.290
poster_translation	3.207	49.265	0.074	0.231	**0.055**	0.133
boxes_6dof	4.014	69.852	**0.143**	0.910	0.230	0.163
boxes_translation	3.853	65.236	**0.158**	0.686	0.187	0.162
hdr_poster	2.774	55.437	**0.322**	0.322	0.373	0.342
hdr_boxes	3.136	55.088	**0.212**	0.597	0.234	0.249

* E = Event, I = IMU, Fr = Figure.

**Table 2:** The root mean squared error (RMSE) of the proposed approach using range (R), events (E) and IMU (I) against using event and IMU.

Sequence	Max Speed (m/s)	Max Mean Optical Flow (Pixel/s)	Length (m)	RMSE (m)	
				REVIO (R + E + I) *	EVIO (E + I)
1	2.089	2210	31.52	0.111	**0.105**
2	2.349	1280	64.89	**0.086**	0.088
3	2.422	1740	55.43	**0.094**	0.109
4	3.489	1557	76.44	**0.091**	0.128

* R = Range, E = Event, I = IMU.

## Data Availability

Not applicable.
